# Psoriasis Is Associated With Myosteatosis but Not Sarcopenia: A Case-Control Study

**DOI:** 10.3389/fmed.2021.754932

**Published:** 2021-10-15

**Authors:** Xiaomei Chen, Hongmei Xiang, Lingling Tan, Jie Zhou, Jing Tang, Xiaoyi Hu, Ming Yang

**Affiliations:** ^1^Department of Dermatology, West China Hospital, Sichuan University, Chengdu, China; ^2^Center of Gerontology and Geriatrics, West China Hospital, Sichuan University, Chengdu, China; ^3^Health Management Center, West China Hospital, Sichuan University, Chengdu, China; ^4^Department of Radiology, West China Hospital, Sichuan University, Chengdu, China; ^5^National Clinical Research Center for Geriatrics, West China Hospital, Sichuan University, Chengdu, China; ^6^Precision Medicine Research Center, West China Hospital, Sichuan University, Chengdu, China

**Keywords:** myosteatosis, muscle wasting, muscle depletion, psoriasis, fat infiltration

## Abstract

**Background:** The link between psoriasis and body fat (or obesity) has been well established. However, there are no reports detailing the possible relationship between psoriasis and fat infiltration in skeletal muscle, also known as myosteatosis. A recent study reported the possible association between psoriasis, arthritis, and sarcopenia (the loss of skeletal muscle mass or function). The present study aimed to explore the possible associations of chronic plaque psoriasis with myosteatosis and sarcopenia.

**Methods:** We conducted a case-control study. In-patients with chronic plaque psoriasis were retrospectively recruited. Healthy controls were prospectively and continuously recruited. Unenhanced cross-sectional chest computed tomography images at the 12th thoracic vertebral level were analyzed using Mimics software. Skeletal muscle area (SMA), skeletal muscle radiodensity (SMD), and intermuscular adiposity tissue (IMAT) were measured. The skeletal muscle index (SMI) was calculated as SMA/height^2^. The percentage of IMAT (IMAT%) was calculated as IMAT/SMA × 100%. Myosteatosis was defined by SMD or IMAT%, whereas sarcopenia was defined by SMI. Propensity score matching was performed to adjust for the main confounders. Logistic regression models were used to evaluate the associations of psoriasis with myosteatosis and sarcopenia.

**Results:** We included 155 psoriasis patients and 512 healthy controls. After propensity score matching, we retained 310 controls. The prevalence of sarcopenia was not significantly different between the psoriasis and control groups (men: 9.8% vs. 14.4%, *p* = 0.244; women: 7.0% vs. 11.7%, *p* = 0.548). Psoriasis patients were more prone to SMD-defined myosteatosis (men: 39.3% vs. 20.8%; women: 46.5% vs. 16.0%; both *p* < 0.001) and IMAT%-defined myosteatosis (men: 21.4% vs. 12.5%, *p* = 0.034; women: 46.5 vs. 28.7%, *p* = 0.042) than the control group. After adjustment for potential confounders, psoriasis was not significantly associated with sarcopenia (odds ratio [OR] 0.51, 95% confidence interval [CI] 0.25–1.19, *p* = 0.136). However, psoriasis was associated with SMD-defined myosteatosis (OR 3.16, 95% CI 1.86–5.37, *p* < 0.001) and IMAT%-defined myosteatosis (OR 1.76, 95% CI 1.04–3.00; *p* = 0.037).

**Conclusions:** Chronic plaque psoriasis is independently associated with myosteatosis but not sarcopenia. Since fat and muscle are considered endocrine organs and can drive the inflammatory process, further studies detailing the interaction between psoriasis, fat, and skeletal muscle are warranted.

## Introduction

Psoriasis is an immune-mediated systemic inflammatory disease that affects the skin and joints (1). According to a recent systematic review, the prevalence of psoriasis varies from 0.5 to 11.4% across study populations (2). Currently, there is a mutual consensus that psoriasis can induce important consequences beyond the skin. It has been associated with many comorbidities, such as obesity, diabetes mellitus, hypertension, metabolic syndrome, depression, and cardiovascular diseases (3, 4). Recently, there has been increasing evidence indicating that psoriasis is linked to body composition, i.e., the percentages of fat and skeletal muscle in human bodies (3).

The relationship between psoriasis and fat has been well documented in the literature (5–7). Fat infiltration in skeletal muscle mass, also known as myosteatosis, includes three components: (i) intermuscular adipose tissue (IMAT), the extracellular fat beneath the fascia and between muscle groups; (ii) intramuscular adipose tissue (IntraMAT), the extracellular fat within an individual muscle; and (iii) intramyocellular lipids (IMCL), the fat stored in droplets in muscle cells (8). Similar to other adipocyte tissues, myosteatosis can produce adipokines, such as leptin, adiponectin, and resistin, which have been reported to mediate the associations between psoriasis, obesity, and cardiovascular diseases (8). These findings imply a possible association between psoriasis and myosteatosis. However, after systematically searching PubMed, Embase, and Web of Science, we failed to identify any study addressing this association.

Conversely, there is limited evidence addressing the association between psoriasis and skeletal muscle (9, 10). A recent case-control study including 51 women with psoriasis arthritis and 44 controls reported that sarcopenia, the loss of muscle mass and muscle strength, was more prevalent in women with psoriatic arthritis than in healthy women. However, no correlation between sarcopenia and psoriatic arthritis activity was observed in the study (10). Notably, psoriatic arthritis accounts for approximately 3% of psoriasis cases, whereas chronic plaque psoriasis, the most common type of psoriasis, accounts for more than 90% of psoriasis cases (11). However, the link between sarcopenia and chronic plaque psoriasis has not yet been reported. Therefore, this study aimed to explore the potential association of chronic plaque psoriasis with sarcopenia and myosteatosis.

## Methods

### Study Population

We conducted a case-control study. First, we retrospectively collected relevant information on adult in-patients with chronic plaque psoriasis in the Dermatology Department of West China Hospital, Sichuan University, from May 2018 to May 2019. A diagnosis of chronic plaque psoriasis was made based on the Chinese guideline for psoriasis (12). The exclusion criteria were as follows: (i) psoriatic arthritis; (ii) visible edema, chronic kidney disease, or any type of tumor; (iii) low-quality computed tomography (CT) images, anatomical distortion (such as chest wall edema), or loss of any muscle mass area on CT images; (iv) missing data on key variables; (v) absence of chest CT images during the hospitalization period; and (vi) treatment with corticosteroids in our department. From August 2019 to July 2020, we prospectively and continuously invited healthy adults without psoriasis who underwent routine health examinations in West China Hospital, Sichuan University, to participate in this study. The exclusion criteria were as follows: (i) absence of chest CT images; (ii) visible edema, chronic kidney disease, or any type of tumor; and (iii) low-quality CT images or any anatomical distortion (such as chest wall edema) or loss of any muscle mass area on CT images. Clinical information collection and blood sampling were performed by trained nurses. The Biomedical Ethics Committee of West China Hospital, Sichuan University approved the study protocol. All prospectively recruited patients signed written informed consent forms.

### CT Image Analysis

CT is used for the direct measurement of skeletal muscle mass and IMAT as well as the indirect estimation of IntraMAT and IMCL by measuring skeletal muscle radiodensity (SMD), which is expressed in Hounsfield units (HUs) (8). The lower the HU, the lower the radiodensity, and the higher the degrees of IntraMAT and IMCL (8). In this study, all chest CT scans were obtained using 16-slice spiral CT scanners (Brilliance; Philips Healthcare, Ohio, USA) with a 5-mm slice thickness in West China Hospital, Sichuan University. The acquisition parameters were as follows: detector collimation of 0.75–1.5 mm, 100–140 kV, and variable mAs based on the individual's body size.

**Figure 1:**
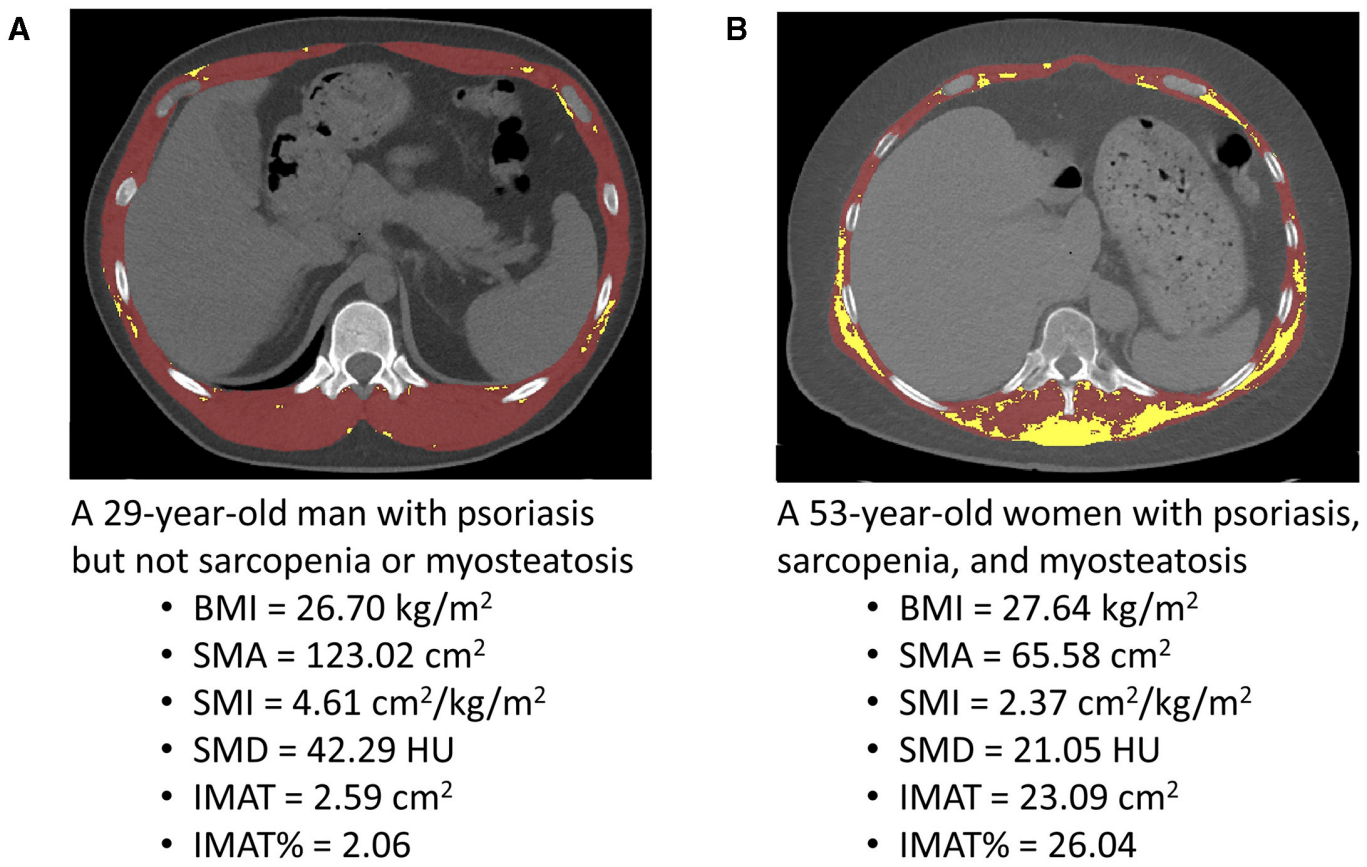
Typical segmented computed tomography (CT) images at the 12th thoracic vertebra level used for the quantification of the skeletal muscle area (SMA), skeletal muscle radiodensity (SMD), and intermuscular adiposity tissue (IMAT) in a young man with psoriasis **(A)** and a middle-aged woman with psoriasis **(B)**. BMI, body mass index; HU, Hounsfield unit; SMI, skeletal muscle index; IMAT%, percentage of IMAT.

We used segmentation software (Mimics version 21.0; Materialise, Leuven, Belgium) to analyze unenhanced cross-sectional CT images at the 12th thoracic vertebral (T12) level. Skeletal muscle mass area (SMA) was segmented according to a widely used muscle tissue threshold CT value, i.e., −29 to +150 HU (13). The muscles at the T12 level include the internus abdominis, erector spinae, obliquus externus, latissimus dorsi, rectus abdominis, and internal and external intercostal muscles (14). The mean SMD of T12 SMA was calculated automatically by Mimics software. The lower the SMD, the higher the degree of myosteatosis. We segmented IMAT according to a widely used threshold of CT value (−30–−190 HU) (15). The higher the IMAT, the higher the degree of myosteatosis (15). A trained researcher (L.T.), who was blinded to the clinical information of the participants, performed all CT image segmentations. Typical segmented chest CT images are presented in Figure 1.

### Definitions of Sarcopenia and Myosteatosis

As previously reported (14, 16), we calculated the skeletal muscle index (SMI) using the equation SMI (cm^2^/m^2^) = SMA (cm^2^)/height^2^ (m^2^). An SMI < 25.75 cm^2^/m^2^ in men or < 20.16 cm^2^/m^2^ in women indicates sarcopenia. Additionally, an SMD < 37.42 HU in men or < 33.17 HU in women indicates SMD-defined myosteatosis. We calculated the percentage of IMAT area at the T12 level (IMAT%) using the equation IMAT% = IMAT/SMA ×100% (17). An IMAT% > 7.51% in men or > 6.83% in women indicates IMAT%-defined myosteatosis (17).

### Covariates

The following clinical information was collected from the healthcare information system: age, sex, body mass index (BMI), smoking status, alcohol consumption status, hypertension, diabetes, and fatty liver. For each patient with psoriasis, body surface area score and Psoriasis Area and Severity Index were evaluated by experienced dermatologists (18). After at least 8 h of fasting, blood was drawn from each participant early in the morning. Fasting glucose, alanine aminotransferase, aspartate aminotransferase, albumin, total cholesterol (TC), triglycerides, high-density lipoprotein-cholesterol (HDL-C), and low-density lipoprotein-cholesterol (LDL-C) were tested according to the standard method, using the Toshiba 200FR Neo analyzer (Toshiba Medical System Co., Ltd., Tokyo, Japan).

### Statistical Analysis

Categorical data were presented as numbers and percentages. Continuous data were presented as the mean and standard deviation (SD) or median and interquartile range where appropriate. Group differences were compared using independent samples *t*-tests or Mann-Whitney *U*-tests for continuous data and Pearson's chi-square tests for categorical data.

Differences between the psoriasis group and the control group were adjusted using propensity score matching analysis (19). Propensity scores were developed using logistic regression based on age, sex, and BMI. The most appropriately matched pairs were identified using a 1:2 nearest neighbor matching algorithm without replacement and with a caliper of 0.05 (19). After matching, group differences were compared again using the abovementioned methods. Additionally, violin plots and box plots were used to present the differences in SMI, SMD, IMAT, and IMAT% between the psoriasis and control groups in men and women. Logistic models were used to explore the associations of psoriasis with sarcopenia, SMD-defined myosteatosis, and IMAT%-defined myosteatosis. Model 1 adjusted for age, sex, and BMI. Model 2 adjusted for age, sex, BMI, smoking status, diabetes, fatty liver, albumin, TC, HDL-C, and LDL-C.

Statistical analyses were performed using R software version 3.5.1 (R Foundation for Statistical Computing, Vienna, Austria) and SPSS software version 26.0 (IBM SPSS Inc., New York, US). A two-sided *p*-value of < 0.05 indicated statistical significance.

## Results

### Study Population

**Figure 2:**
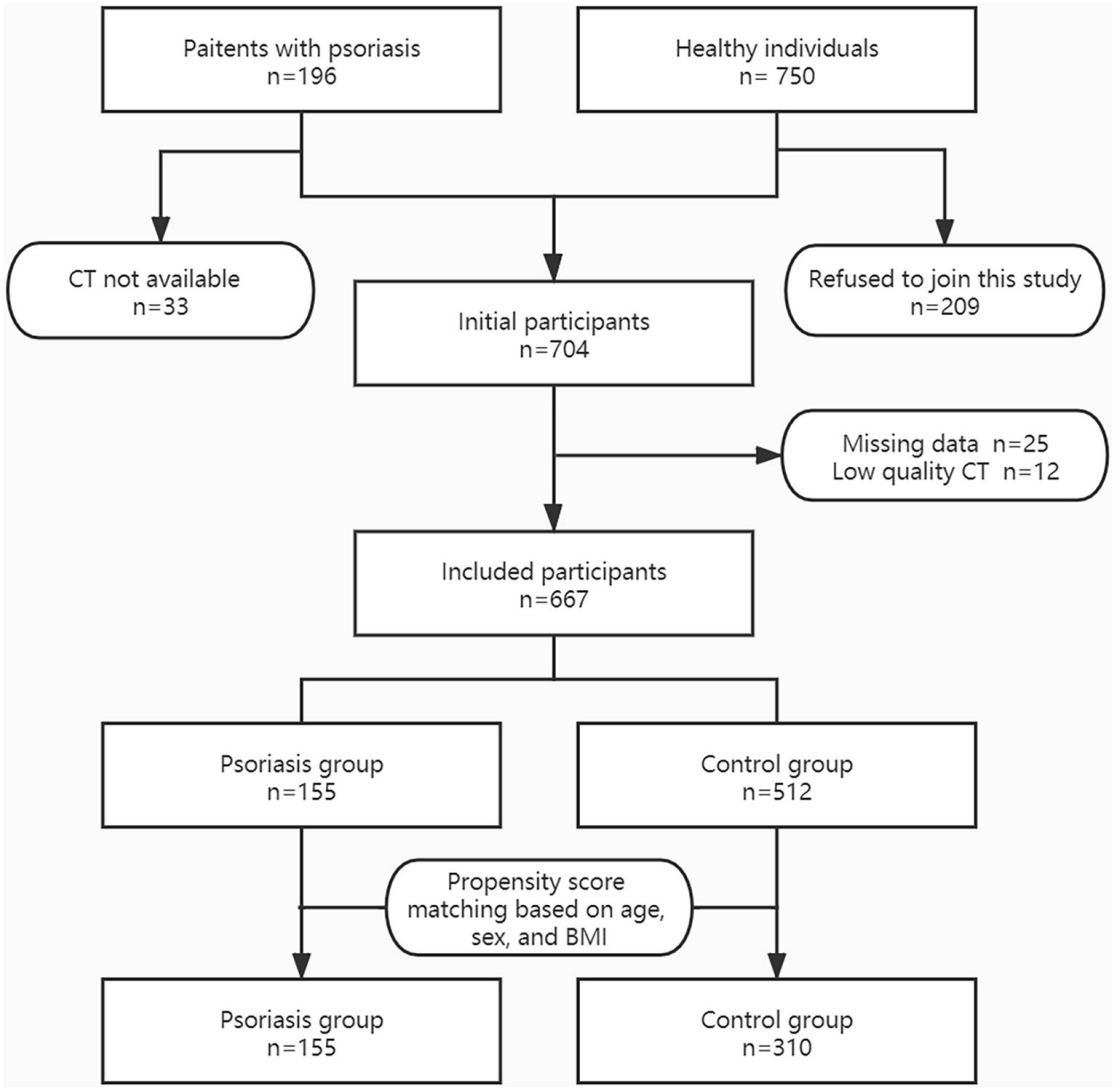
Flow chart of the study population. BMI, body mass index; CT, computed tomography.

Figure 2 shows the flow chart of the study population selection. Initially, we included 196 patients with psoriasis, of whom 33 were excluded because chest CT result was not available. We continuously invited 750 healthy adults, of whom 209 refused to join this study. Furthermore, 37 participants were excluded due to missing data or low-quality CT images. Consequently, we included 155 patients in the psoriasis group and 512 individuals in the control group.

Table 1 shows the characteristics of the study population. Individuals in the psoriasis group were significantly older than those in the control group (45.2 vs. 42.9 years, *p* = 0.031). The mean BMI was significantly higher in the psoriasis group than in the control group (24.41 vs. 23.20, *p* < 0.001). Additionally, the psoriasis group had fewer women than the control group, although the difference was not statistically significant (27.7 vs. 35.2%, *p* = 0.086). Therefore, we adjusted for age, sex, and BMI using propensity score matching in the following analysis.

**Table 1:** Characteristics of the study population.

**Characteristic**	**Control** **(*n* = 512)**	**Psoriasis** **(*n* = 155)**	***p*-value**
Age, years, mean (SD)	42.9 (10.7)	45.2 (13.1)	0.031
Women, *n* (%)	180 (35.2)	43 (27.7)	0.086
Smoker, *n* (%)	136 (26.6)	76 (49.0)	<0.001
Alcohol drinker, *n* (%)	153 (29.9)	43 (27.9)	0.640
**Comorbidities**, ***n*** **(%)**			
Hypertension	64 (12.5)	20 (12.9)	0.895
Diabetes	22 (4.3)	9 (5.8)	0.434
Fatty liver	31 (6.1)	22 (14.2)	0.001
BSA score, median (IQR)	–	32.0 (33.5)	–
PASI score, median (IQR)	–	23.1 (21.0)	–
**PASI groups**			
<7	–	14 (9.0)	–
7–12	–	15 (9.7)	–
>12	–	126 (81.3)	–
BMI, kg/m^2^, mean (SD)	23.20 (3.00)	24.41 (3.21)	<0.001
Fasting glucose, mmol/l, mean (SD)	5.10 (1.19)	5.06 (1.14)	0.689
ALT, IU/l, median (IQR)	19.0 (16.0)	22.0 (17.0)	0.049
AST, IU/l, median (IQR)	21.0 (9.0)	21.0 (7.0)	0.497
Albumin, g/l, mean (SD)	47.94 (2.45)	43.32 (4.67)	<0.001
TC, mmol/l, mean (SD)	4.92 (0.88)	4.51 (1.03)	<0.001
TG, mmol/l, mean (SD)	1.58 (1.21)	1.81 (1.27)	0.035
HDL-C, mmol/l, mean (SD)	1.41 (0.36)	1.21 (0.38)	<0.001
LDL-C, mmol/l, mean (SD)	2.91 (.077)	2.81 (1.21)	0.259
**Body composition variables**			
SMA, cm^2^, mean (SD)	93.33 (21.64)	93.12 (20.92)	0.917
SMI, cm^2^/m^2^, mean (SD)	4.08 (1.08)	3.83 (0.80)	0.007
SMD, HU, mean (SD)	40.33 (4.99)	36.97 (6.56)	<0.001
IMAT, cm^2^, median (IQR)	2.93 (3.34)	4.01 (4.53)	0.004
IMAT%, median (IQR)	3.21 (3.47)	4.35 (4.43)	0.002

*ALT, alanine aminotransferase; AST, aspartate aminotransferase; BMI, body mass index; BSA, body surface area; HDL-C, high-density lipoprotein-cholesterol; HU, Hounsfield Unit; IMAT, intermuscular adiposity tissue, IMAT%, percentage of intermuscular adiposity tissue; IQR, interquartile range; LDL-C, low-density lipoprotein-cholesterol; PASI, psoriasis area and severity index; SD, standard deviation; SMA, skeletal muscle cross-sectional area; SMD, skeletal muscle radiodensity; SMI, skeletal muscle index (skeletal muscle cross-sectional area/BMI); TC, total cholesterol; TG, triglycerides*.

### Group Differences After Propensity Score Matching

After propensity score matching, we retained 310 cases in the control group. Supplementary Figure 2 shows the jitter plot of propensity score distributions. Table 2 shows the characteristics of the study population after propensity score matching. Age and BMI were no longer significantly different between the psoriasis group and the control group in both men and women. Compared with their counterparts, psoriasis patients had significantly lower albumin and TC levels in men and women. Furthermore, men with psoriasis had a significantly higher frequency of fatty liver and lower HDL-C and LDL-C levels than their healthy counterparts. These differences were not statistically significant in women (Table 2).

**Table 2:** Characteristics of the study population after propensity score matching.

**Characteristic**	**Men (*****n*** **= 328)**			**Women (*****n*** **= 137)**		
	**Control (*n* = 216)**	**Psoriasis (*n* = 112)**	***p*-value**	**Control (*n* = 94)**	**Psoriasis (*n* = 43)**	***p*-value**
Age, years, mean (SD)	45.5 (10.3)	44.7 (12.9)	0.516	44.7 (11.2)	46.5 (13.8)	0.428
Smoker, *n* (%)	66 (30.6)	74 (66.1)	<0.001	8 (8.5)	2 (4.7)	0.725
Alcohol drinker, *n* (%)	78 (36.1)	41 (36.9)	0.883	10 (10.6)	4 (9.3)	0.811
Comorbidities, *n* (%)						
Hypertension	38 (17.6)	17 (15.2)	0.579	10 (10.6)	3 (7.0)	0.497
Diabetes	10 (4.6)	7 (6.3)	0.530	4 (4.3)	2 (4.7)	0.916
Fatty liver	7 (7.9)	17 (15.2)	0.039	6 (6.4)	5 (11.6)	0.320
BMI, kg/m^2^, mean (SD)	24.60 (2.92)	24.54 (3.18)	0.858	23.58 (2.66)	24.06 (3.28)	0.361
Fasting glucose, mmol/l, mean (SD)	5.22 (1.15)	4.98 (0.94)	0.062	5.22 (1.14)	5.26 (1.55)	0.854
ALT, IU/l, median (IQR)	20.0 (17.0)	22.0 (17.0)	0.458	19.5 (15.0)	20.0 (19.0)	0.884
AST, IU/l, median (IQR)	21.0 (9.0)	20.5 (7.0)	0.061	21.0 (9.0)	21.0 (9.0)	0.980
Albumin, g/l, mean (SD)	47.70 (2.28)	43.51 (4.52)	<0.001	48.20 (2.72)	42.85 (5.08)	<0.001
TC, mmol/l, mean (SD)	5.02 (0.87)	4.55 (1.00)	<0.001	4.99 (0.86)	4.41 (1.10)	0.001
TG, mmol/l, mean (SD)	1.70 (1.25)	1.80 (1.21)	0.552	1.79 (1.65)	1.88 (1.44)	0.745
HDL-C, mmol/l, mean (SD)	1.39 (0.37)	1.14 (0.30)	<0.001	1.40 (0.38)	1.39 (0.50)	0.887
LDL-C, mmol/l, mean (SD)	2.99 (0.76)	2.80 (0.80)	0.041	2.88 (0.72)	2.85 (1.92)	0.894
Sarcopenia	31 (14.4)	11 (9.8)	0.244	11 (11.7)	3 (7.0)	0.548
SMD-defined myosteatosis	45 (20.8)	44 (39.3)	<0.001	15 (16.0)	20 (46.5)	<0.001
IMAT%-defined myosteatosis	27 (12.5)	24 (21.4)	0.034	27 (28.7)	20 (46.5)	0.042

*ALT, alanine aminotransferase; AST, aspartate aminotransferase; BMI, body mass index; BSA, body surface area; HDL-C, high-density lipoprotein-cholesterol; HU, Hounsfield Unit; IMAT, intermuscular adiposity tissue, IMAT%, percentage of intermuscular adiposity tissue; IQR, interquartile range; LDL-C, low-density lipoprotein-cholesterol; PASI, psoriasis area and severity index; SD, standard deviation; SMA, skeletal muscle cross-sectional area; SMD, skeletal muscle radiodensity; SMI, skeletal muscle index (skeletal muscle cross-sectional area/BMI; TC, total cholesterol; TG, triglycerides)*.

**Figure 3:**
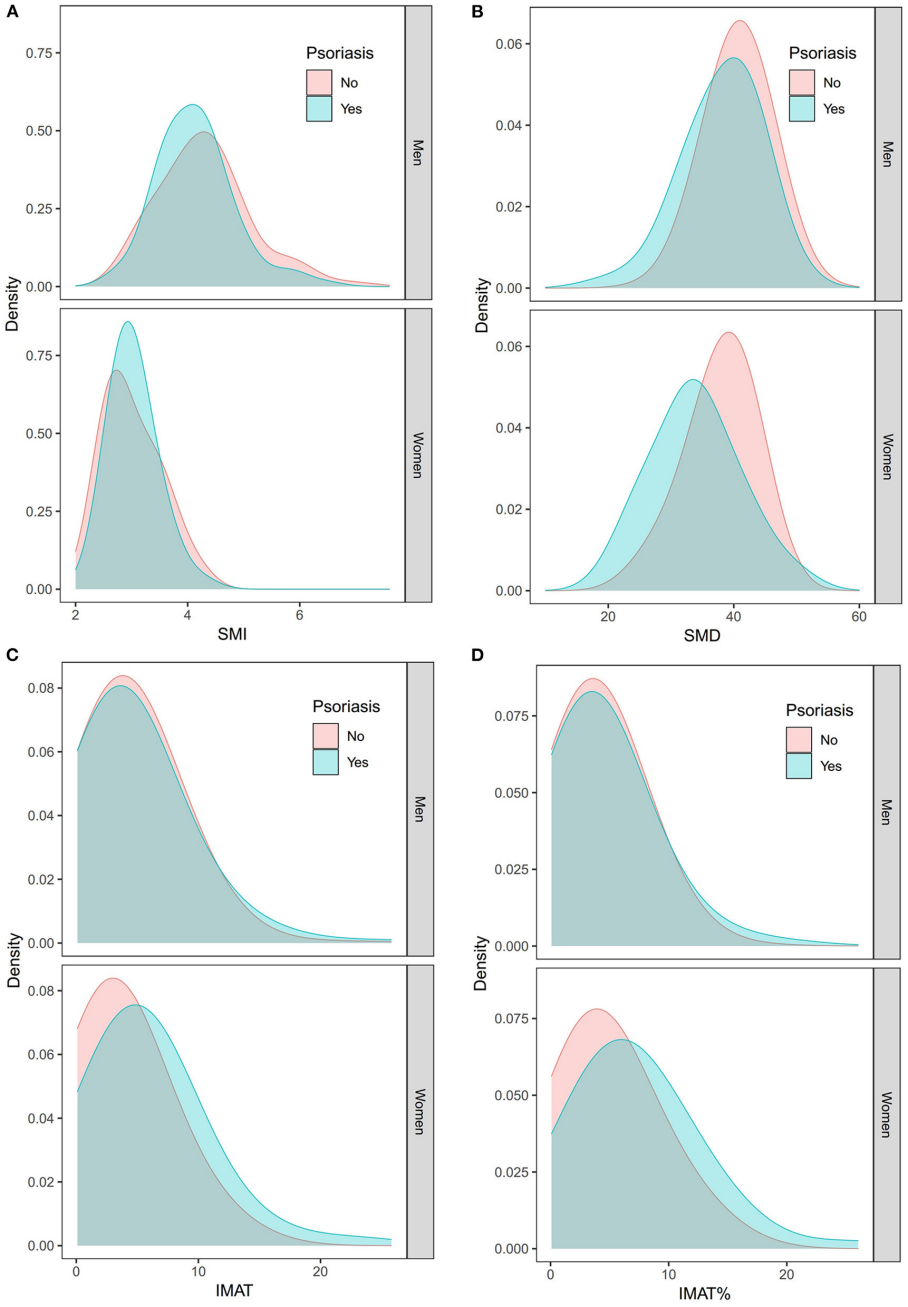
Density distributions of the SMI **(A)**, SMD **(B)**, IMAT **(C)**, and IMAT% **(D)** in the psoriasis group and the control group stratified by sex. IMAT, intermuscular adiposity tissue; IMAT%, percentage of IMAT; SMD, skeletal muscle radiodensity; SMI, skeletal muscle index.

**Figure 4:**
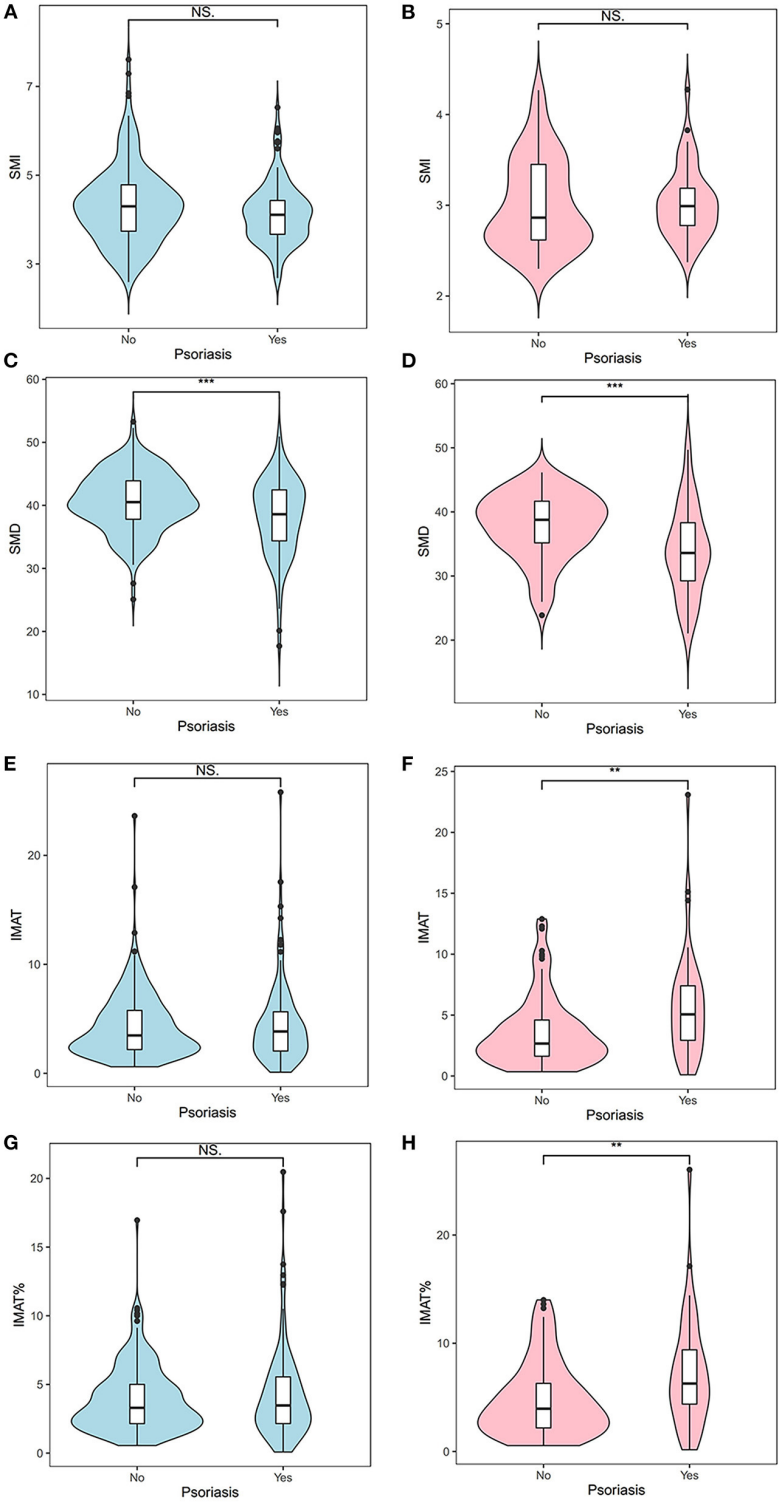
Differences between the psoriasis group and the control group with respect to the SMI **(A,B)**, SMD **(C,D)**, IMAT **(E,F)**, and IMAT% **(G,H)**. Blue indicates men; pink indicates women. **: < 0.010; ***: < 0.001. IMAT, intermuscular adiposity tissue; IMAT%, percentage of IMAT; NS, not significant; SMD, skeletal muscle radiodensity; SMI, skeletal muscle index.

Figure 3 shows the density distributions of SMI, SMD, IMAT, and IMAT% in the psoriasis group and the control group, stratified by sex. As shown in Figure 4, the mean SMI was not significantly different between the psoriasis and control groups. However, psoriasis patients had a significantly lower mean SMD than the controls in men and women (both *p* < 0.001). Moreover, psoriasis patients had significantly higher mean IMAT and IMAT% than the control group in women (both *p* < 0.010). However, neither IMAT nor IMAT% was significantly different between the groups in men (both *p* > 0.05).

### Associations of Sarcopenia and Myosteatosis With Psoriasis

As shown in Table 2, the prevalence of sarcopenia was not significantly different between the psoriasis and control groups (men: 9.8 vs. 14.4%, *p* = 0.244; women: 7.0 vs. 11.7%, *p* = 0.548). However, compared with their counterparts, psoriasis patients were more prone to SMD-defined myosteatosis (men: 39.3 vs. 20.8%; women: 46.5 vs. 16.0%; both *p* < 0.001) and IMAT%-defined myosteatosis (men: 21.4 vs. 12.5%, *p* = 0.034; women: 46.5 vs. 28.7%, *p* = 0.042).

Moreover, after adjustment for potential confounders, psoriasis was not significantly associated with sarcopenia (odds ratio [OR] 0.51, 95% confidence interval [CI] 0.25–1.19; *p* = 0.136). However, psoriasis was associated with SMD-defined myosteatosis (OR 3.16, 95% CI 1.86–5.37; *p* < 0.001) and IMAT%-defined myosteatosis (OR 1.76, 95% CI 1.04–3.00; *p* = 0.037) (Table 3).

**Table 3:** Association of psoriasis with sarcopenia or myosteatosis by logistic regression analysis in the study population after propensity score matching.

	**OR**	**95% CI**	***p*-value**
**Sarcopenia**			
Psoriasis (unadjusted)	0.63	0.34–1.20	0.161
Psoriasis (adjusted Model 1)	0.60	0.32–1.16	0.128
Psoriasis (adjusted Model 2)	0.51	0.25–1.19	0.136
**SMD-defined myosteatosis**			
Psoriasis (unadjusted)	2.93	1.91–4.49	<0.001
Psoriasis (adjusted Model 1)	3.71	2.25–6.11	<0.001
Psoriasis (adjusted Model 2)	3.16	1.86–5.37	<0.001
**IMAT%-defined myosteatosis**			
Psoriasis (unadjusted)	1.88	1.19–2.97	0.007
Psoriasis (adjusted Model 1)	2.00	1.21–3.28	0.006
Psoriasis (adjusted Model 2)	1.76	1.04–3.00	0.037

*BMI, body mass index; CI, confidence interval; IMAT%, percentage of intermuscular adiposity tissue; OR, odds ratio; SMD, skeletal muscle radiodensity*.

*Model 1: adjusted for age, sex, and BMI*.

*Model 2: further adjusted for smoking, diabetes, fatty liver, albumin, total cholesterol, high-density lipoprotein-cholesterol, and low-density lipoprotein-cholesterol based on Model 1*.

## Discussion

To the best of our knowledge, this is the first study to explore the association between chronic plaque psoriasis and myosteatosis. Our study demonstrated that myosteatosis, regardless of whether it is defined by SMD or IMAT%, was independently associated with psoriasis. There is no established molecular mechanism to explain the association between psoriasis and myosteatosis. However, cytokines produced by adipose tissue, also known as adipokines, may play an important role in this link (20, 21). For example, IMAT can produce leptin, a proinflammatory adipokine, which increases the production of T helper 1 cytokines and IL-17A (20, 22, 23). These cytokines have a crucial role in psoriasis pathogenesis (20, 22, 23).

BMI-defined obesity has been well recognized as an independent risk factor for psoriasis (5, 24). For example, a systematic review of 16 studies reported a pooled OR for the association between psoriasis and BMI-defined obesity of 1.66 (95% 1.46–1.89) (25). Additionally, there is evidence indicating that obesity is associated with the incidence of psoriasis (4). Adipose tissues in obese people have been proven to promote the development of Th17 cells (26). IL-17A expression was also upregulated in obesity (27). Moreover, psoriasis has been associated with increased inflammatory cytokines, in which the IL-23/Th17 axis with TNF-α, IL-21, and IL-22 play a role in the control of inflammation (28). This might partly explain the association between obesity and psoriasis. However, our study identified an independent association between psoriasis and myosteatosis even after adjusting for BMI. Therefore, this association might not be fully explained by the presence of obesity.

The diagnostic criteria of myosteatosis have not yet been established; however, SMD and IMAT are the two most frequently used indicators to diagnose myosteatosis (29, 30). According to a recent systematic review, among the 125 included studies, 82 (65.6%) and 27 (21.6%) studies used IMAT and SMD to define myosteatosis, respectively (31). In this study, we diagnosed myosteatosis based on SMD and IMAT. It is not known whether SMD or IMAT is better for defining myosteatosis. However, a retrospective study found that a lower SMD (but not a higher IMAT) was significantly associated with a higher risk of 6-month mortality in ICU patients (32). Another study demonstrated that a lower SMD (but not a higher IMAT) was independently associated with the rate of torque development and peak knee extension in older adults (33). These findings imply that SMD is a better indicator for myosteatosis; however, there is a need for stronger evidence, especially from prospective cohort studies.

A small case-control study reported a higher prevalence of sarcopenia, defined by appendicular lean mass *via* bioimpedance analysis, in women with psoriatic arthritis (13.7%) compared to healthy women (9%). However, the relevant *p*-value was not reported in the study (10). Our finding that psoriasis was not associated with sarcopenia is inconsistent with the findings of the aforementioned study. There are several possible reasons. First, the mean age of our psoriasis patients (45.2 years) was lower than that in the other two studies (65.6 and 64.1 years, respectively) (9, 10). As sarcopenia is an age-related muscle disease (34), the prevalence of sarcopenia in our study population was low. Thus, group differences regarding sarcopenia may not be detected. Second, the methods for measuring muscle mass and the diagnostic criteria for sarcopenia varied across these studies. Finally, the study populations were also heterogeneous. Our patients were hospitalized and had severe sarcopenia, whereas the patients in the other two studies lived in the community.

Notably, we defined sarcopenia based on skeletal muscle mass in the present study. In the literature, two major opinions about the definition of sarcopenia exist. Some researchers argue that sarcopenia should be defined according to low skeletal muscle mass and low muscle function (strength and/or physical performance), using the EWGSOP criteria (34), whereas others believe that sarcopenia should be defined “using only muscle mass” (35). It would be interesting to explore the association between psoriasis and muscle strength in the future.

Our study has some limitations. First, the psoriasis group was recruited retrospectively in a single center; therefore, selection bias might have been introduced, and our study population may be underrepresented. Second, because of the case-control design, the causal relationship between psoriasis and myosteatosis could not be determined. Third, we did not explore the possible associations of the severity of psoriasis with sarcopenia and myosteatosis, as most of the patients (81.3%) had severe psoriasis. However, a recent systematic review found conflicting data concerning the association between psoriasis severity and body composition parameters (3). Therefore, further studies with a large sample size are required. Fourth, some important confounders, including daily exercise, physical function, dietary nutrient intake, familial dyslipidemia, polycystic ovary syndrome, and medications were not adjusted for in our logistic models. Fifth, we did not define myosteatosis *via* histology, which is supposed to be the “gold standard.” However, the invasive feature of muscle biopsy does not make it an appropriate procedure for patients with psoriasis. Finally, we did not measure and adjust for inflammatory markers, such as C-reactive protein and IL-6, which were supposed to be associated with sarcopenia and psoriasis (36, 37).

## Conclusion

Our study indicates that chronic plaque psoriasis is independently associated with myosteatosis; however, it is not independently associated with sarcopenia. Since fat and muscle are considered endocrine organs and can drive the inflammatory process, future studies detailing the interactions between psoriasis, fat, and skeletal muscle are warranted. Moreover, it would be interesting to design prospective cohort studies to explore the causal relationships between these conditions.

## Data Availability Statement

The raw data supporting the conclusions of this article will be made available by the authors, without undue reservation.

## Ethics Statement

The studies involving human participants were reviewed and approved by Biomedical Ethics Committee of West China Hospital, Sichuan University. The patients/participants provided their written informed consent to participate in this study.

## Author Contributions

XC and MY: conception and design of study. LT: imaging data analysis. XC, XH, and MY: drafting the manuscript. MY: statistical analysis. XC, HX, LT, JZ, JT, XH, and MY: acquisition, analysis, interpretation of data, and critical revision of the manuscript for important intellectual content. All authors contributed to the article and approved the submitted version.

## Funding

This work was supported by the Health Commission of Sichuan Province (Grant Number: 17ZD022) and the K&D Program of the Sichuan Science and Technology Department (Grant Number: 2020YFS0573). The sponsor had no role in the design and conduct of this study.

## Conflict of Interest

The authors declare that the research was conducted in the absence of any commercial or financial relationships that could be construed as a potential conflict of interest.

## Publisher's Note

All claims expressed in this article are solely those of the authors and do not necessarily represent those of their affiliated organizations, or those of the publisher, the editors and the reviewers. Any product that may be evaluated in this article, or claim that may be made by its manufacturer, is not guaranteed or endorsed by the publisher.

## Abbreviations

IMAT: intermuscular adiposity tissue
SMD: skeletal muscle radiodensity
SMI: skeletal muscle index
CT: BMI body mass index
HDL-C: high-density lipoprotein cholesterol
LDL-C: low-density lipoprotein cholesterol
SMA: skeletal muscle area
IntraMAT: intramuscular adipose tissue
IMCL: intramyocellular lipids
HU: Hounsfield unit.
