# Supplementary material for: Psoriasis Is Associated With Myosteatosis but Not Sarcopenia: A Case-Control Study
Source: Front Med (Lausanne). 2021 Oct 15;8:754932. doi: 10.3389/fmed.2021.754932 (PMC8554055; doi:10.3389/fmed.2021.754932)
Supplement: Supplementary file 1 [file Data_Sheet_1.PDF]

Psoriasis is associated with myosteatosis but not sarcopenia: a case-control study

Xiaomei Chen [a], Hongmei Xiang [a], Lingling Tan [b], Jie Zhou [c], Jing Tang [d], Xiaoyi Hu [b, c, *], Ming Yang [b, e, f, *]

[a] Department of Dermatology, West China Hospital, Sichuan University, Chengdu, China

[b] Center of Gerontology and Geriatrics, West China Hospital, Sichuan University, Chengdu, China

[c] Health Management Center, West China Hospital, Sichuan University, Chengdu, China

[d] Department of Radiology, West China Hospital, Sichuan University, Chengdu, China

[e] National Clinical Research Center for Geriatrics, West China Hospital, Sichuan University, Chengdu, China

[f] Precision Medicine Research Center, West China Hospital, Sichuan University, Chengdu, China

***Corresponding Author:**

Ming Yang

National Clinical Research Center for Geriatrics, West China Hospital, Sichuan University, No. 37 Guoxue Lane, Chengdu, Sichuan 610041, China

Telephone: +86 28 85422321

Fax: +86 28 85422321

Email: yanmgier@gmail.com

Xiaoyi Hu

Center of Gerontology and Geriatrics, West China Hospital, Sichuan University, No. 37 Guoxue Lane, Chengdu, Sichuan 610041, China

Telephone: +86 28 85422326

Fax: +86 28 85422326

Email: 6913570@qq.com

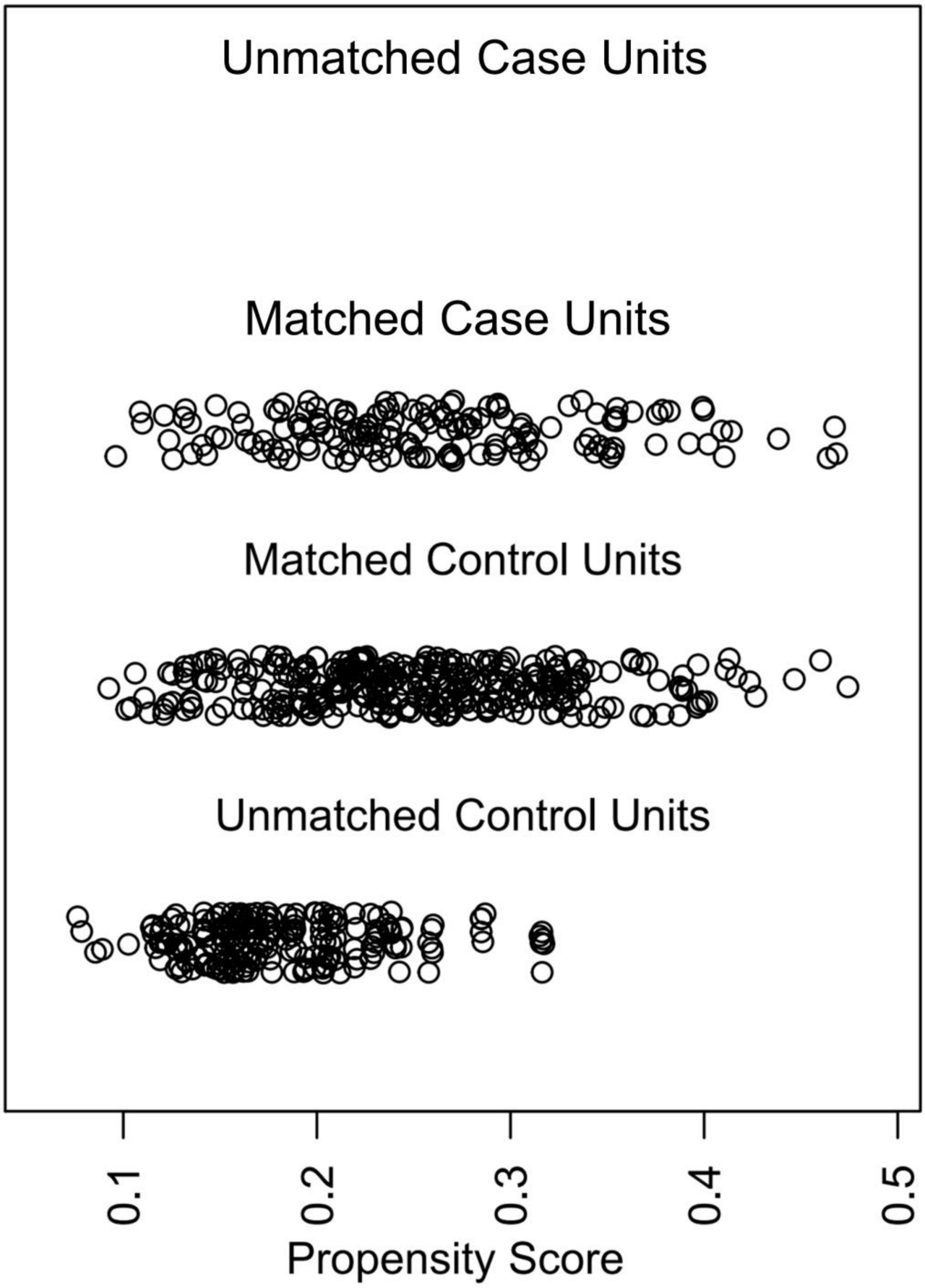

Supplementary Figure 1. Jitter plot of propensity score distributions
